# Gas-Transport Characteristics of PdCu–Nb–PdCu Membranes Modified with Nanostructured Palladium Coating

**DOI:** 10.3390/ijms23010228

**Published:** 2021-12-25

**Authors:** Iliya Petriev, Polina Pushankina, Nikita Shostak, Mikhail Baryshev

**Affiliations:** 1 Department of Physics, Kuban State University, 350040 Krasnodar, Russia; polina_pushankina@mail.ru (P.P.); baryshev_mg@mail.ru (M.B.); 2 Laboratory of Problems of Stable Isotope Spreading in Living Systems, Southern Scientific Centre of the RAS, 344000 Rostov-on-Don, Russia; 3 Department of Oil and Gas Business, Kuban State Technological University, 350040 Krasnodar, Russia; shostak@kubstu.ru

**Keywords:** fifth group metals, composite membranes, hydrogen permeability, nanostructured surface, catalytic layer, high-purity hydrogen

## Abstract

A method for obtaining composite gas-diffusion PdCu–Nb–PdCu membranes modified with a nanostructured crystalline coating was developed to increase the performance of Nb-based membranes. A modifying functional layer with a controlled size and composition was synthesized by electrochemical deposition, which made it possible to determine a certain geometric shape for palladium nanocrystallites. Developed PdCu–Nb–PdCu membranes have demonstrated flux values up to 0.232 mmol s^−1^ m^−2^ in the processes of diffusion purification of hydrogen at 400 °C. A very significant difference in the hydrogen fluxes through the modified and non-modified composite PdCu–Nb–PdCu membranes reached 1.73 times at the lower threshold temperature of 300 °C. Cu doping of protective layer did not affect the selective properties of the membranes, which was confirmed by the obtained high selectivity values up to 1323, and made it possible to reduce the noble metal content. The research data indicate that the modification of the membrane surface significantly accelerates the hydrogen transfer process at sufficiently low temperatures due to the acceleration of dissociative–associative processes on the surface. The reported approach demonstrates new possibilities for creating productive and cost-efficient membranes based on niobium.

## 1. Introduction

The active development of hydrogen energy and the deliberate creation of hydrogen clusters in a number of countries are caused by a clean hydrogen burning reaction and quite a wide range of applications of hydrogen in industries ranging from petrochemistry for the production of hydrocarbon fuels to microelectronics [1,2,3,4]. This increases the demand for the use and, hence, the production, of high-purity hydrogen. Today, the most demanded and promising technology for producing high-purity hydrogen is steam reforming with membrane separation, since it requires a minimum number of interactions during operation and low amounts of energy [5]. Therefore, the actual scientific task is the theoretical and experimental investigation of different membrane processes [6,7,8], in particular the development of the highly efficient membrane, hydrogen. Palladium and its alloys are often used as the basis of these hydrogen-permeable membranes, as palladium has high permeability and selectivity with respect to hydrogen [9,10,11]. However, the high cost of such membranes and the tendency to embrittlement in a hydrogen environment are serious disadvantages. According to recent studies [12,13], group V metals are a promising and fairly inexpensive replacement for palladium to manufacture hydrogen-permeable membranes on their basis. Since group V metals have a body-centered cubic (BCC) lattice, they are highly permeable to hydrogen, i.e., have high hydrogen solubility and rapid diffusion [14]. This property speeds up the transport of hydrogen through membranes by several times, compared to palladium membranes.

However, there are a number of challenges that prevent the use of pure group V metals. Thus, the high solubility of hydrogen in these metals is both a positive and a negative characteristic because it can cause metal crystal lattice saturation with hydrogen atoms and create an unacceptably high concentration of dissolved hydrogen, which subsequently leads to mechanical destruction of the membrane during operation [15,16]. The solution to this problem is the alloying of group V metals with other metals. For example, in [17,18], a higher flux and mechanical strength are investigated in membranes based on the alloys of niobium with W, Ru, and Mo and compared to pure niobium.

Another problem which has not yet been fully resolved is the formation of surface oxide layers that block the dissociative adsorption and associative desorption of hydrogen molecules, thereby preventing their permeation through the membrane. This problem can be eliminated by applying a thin film of palladium or its alloys on both sides of the niobium membrane [14,19]. However, the deposition of such a layer does not allow to achieve the maximum permeability in operating conditions, which can be provided by their high rate of transcrystalline transfer provided by the structural features of the metals of the group V. Hydrogen transport at low pressures up to 0.1 MPa is controlled mainly by surface processes [15,20], but some articles [13,21] have indicated that dissociative–associative processes also have a significant effect on permeability at high pressures. In these articles, the experimental hydrogen fluxes were approximately 1.5 times lower than expected. Hence, it can be concluded that the formation of a classical smooth palladium layer on the niobium membranes surface does not provide the maximum possible permeability of niobium under operating conditions, since it does not remove all surface limitations. In this case, one way to increase hydrogen recovery may be to increase the active surface area of the membrane by applying a modifying nanostructured coating [22,23,24]. Such solution can play a positive role in the acceleration of dissociative–associative processes on the surface, significantly increasing the hydrogen permeability [25,26,27].

Taking into account the above, the aim of this article is the acceleration of the surface stages of hydrogen transport through the composite PdCu–Nb–PdCu membrane by applying a surface-modifying coating based on palladium nanocrystallites. Such membranes, currently being developed, can become an effective basis for hydrogen diffusion purification devices used in the electrolytic hydrogen isotopes separation processes [28,29], etc.

## 2. Results and Discussion

**Figure 1:**
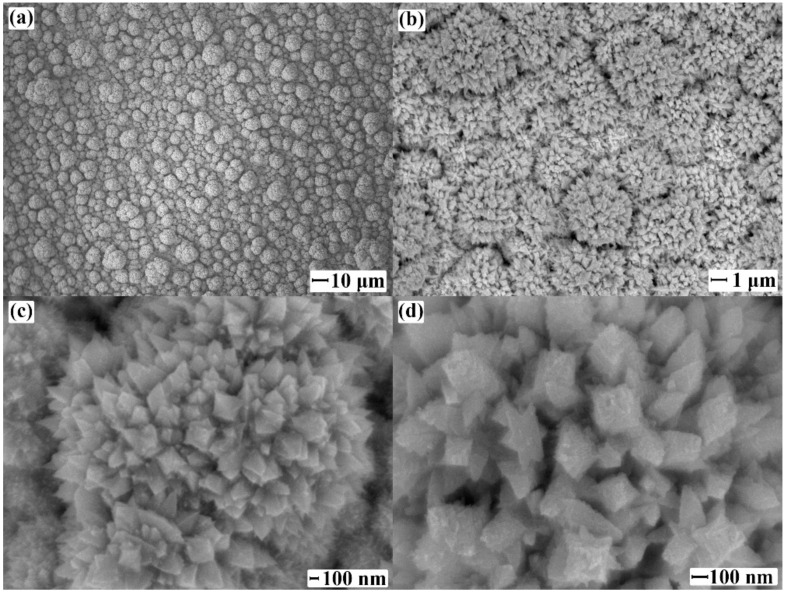
Microphotographs of the PdCu–Nb–PdCu films surface modified with palladium nanocrystallites at a magnification of 500 (**a**), 5000 (**b**), 30,000 (**c**), 50,000 (**d**).

Microphotographs of the synthesized nanostructured modifying coating are shown in Figure 1. SEM images demonstrate the crystalline bipyramidal structure of the particles. The obtained palladium nanocrystallites on average belong to the size range of 110–170 nm.

The analysis and calculations using the obtained microphotographs of the samples of PdCu–Nb–PdCu films were carried out in the modular program Gwyddion (Table 1). According to the calculations, the active surface area of the composite PdCu–Nb–PdCu foil modified with a nanocrystalline coating and increased by 10.5 times relative to the non-modified foil; the roughness coefficient was 16.18.

**Figure 2:**
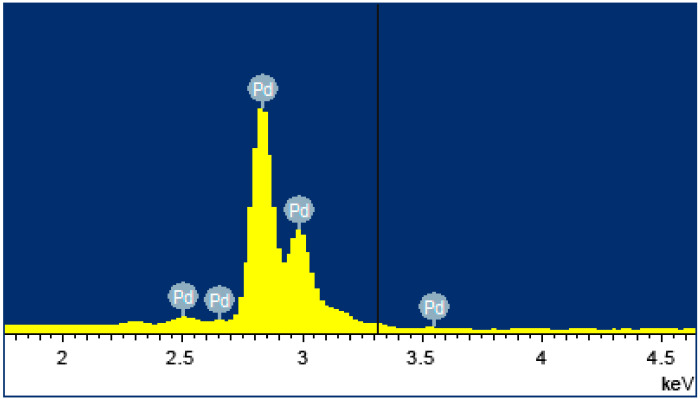
Elemental composition spectrum of the modified PdCu–Nb–PdCu film sample.

The elemental composition of the nanostructured modifying coating was investigated on a semiconductor energy dispersive attachment INCA (Oxford) as part of a JEOL JSM-7500F scanning electron microscope. Figure 2 shows the resulting elemental composition spectrum, according to which the content of palladium in the surface layer was 95.76%.

**Figure 3:**
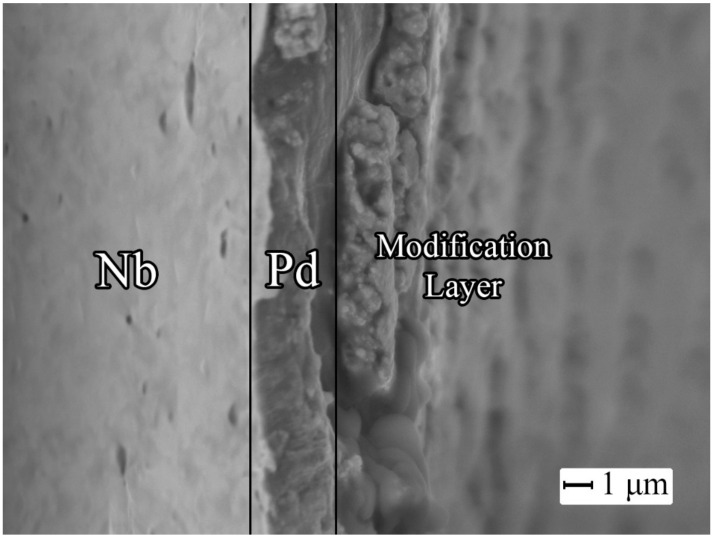
Microphotograph of a modified PdCu–Nb–PdCu film section.

The thicknesses of protective Pd–Cu and catalytic nanostructured palladium layers deposited on niobium foil were estimated using microphotographs of sections taken on a JEOL JSM-7500F scanning electron microscope. As shown in Figure 3 data, the thickness of the smooth protective layer was 1.1 μm, the thickness of the modifying nanostructured layer was 0.8 μm.

**Figure 4:**
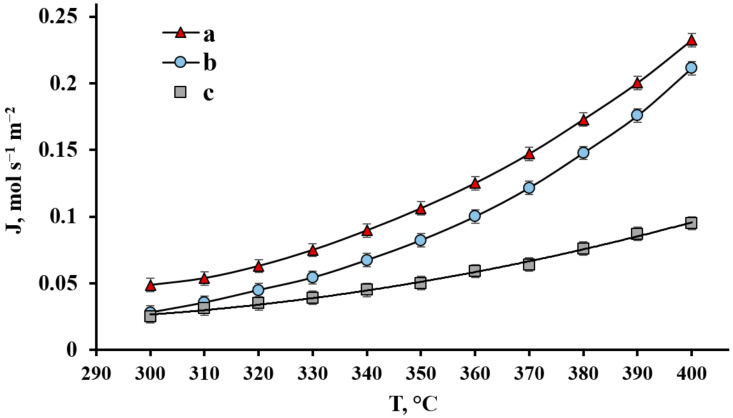
Temperature dependence of the hydrogen flux at Δ*p* = 0.1 MPa through a PdCu–Nb–PdCu membrane modified with a nanostructured coating (a) and non-modified membrane (b) and a regular Pd membrane (c).

The experiment on the modified PdCu–Nb–PdCu membranes gas-transport parameters consisted of two steps. In both experiments, developed modified and non-modified PdCu–Nb–PdCu membranes have been compared to a regular pure Pd membrane, which is a basis for commercially available membranes used in industry. The first step is temperature measurements of the hydrogen flux through the developed membranes at a constant gauge pressure ΔP = 0.1 MPa. The temperature dependence of the hydrogen flux through a composite PdCu–Nb–PdCu membrane modified with a nanocrystalline coating and a membrane without modification is shown in Figure 4. Usually, the membranes based on the group V metals are used at temperatures close to 400 °C [13,15,21]; therefore, considering that the effect of the modification with such that a coating is most expected in the low temperature range, the experiment was carried out in the temperature range from 300 °C to 400 °C. In addition, the membrane thermal degradation caused by interdiffusion between niobium and palladium coating slows down significantly in this range [20,30]. Thus, an operating temperature decrease can significantly increase the membrane durability.

According to the obtained experimental data shown in Figure 4, the hydrogen flux through a PdCu–Nb–PdCu membrane modified with a nanocrystalline coating at a gauge pressure of 0.1 MPa and a temperature of 300 °C was 0.049 mol s^−1^ m^−2^, which is 1.73 times higher than the flux through a non-modified smooth membrane of the same composition. This result can most likely be achieved only in the case of limiting the hydrogen transfer by surface processes, since surface modification is unlikely to affect the diffusion process in the metal foil bulk. The deposition of a nanostructured, highly developed functional layer leads to an increase in the membrane surface roughness and in the active centers number. The high concentration of such catalytic centers is probably due to the growth features for the nanocrystallite’s geometric shape. The hydrogen flux of a PdCu–Nb–PdCu membrane modified with such coating reached values up to 0.232 mol s^−1^ m^−2^ at the upper threshold temperature of 400 °C. However, the hydrogen flux values of the modified and non-modified PdCu–Nb–PdCu membranes ratio decreased, which demonstrates that flux only increase by a factor of 1.1. This effect can be explained by the fact that the contribution of surface processes to the permeability limitation decreases with an increase in temperature; therefore, the developed nanostructured coating effect is minimized.

**Figure 5:**
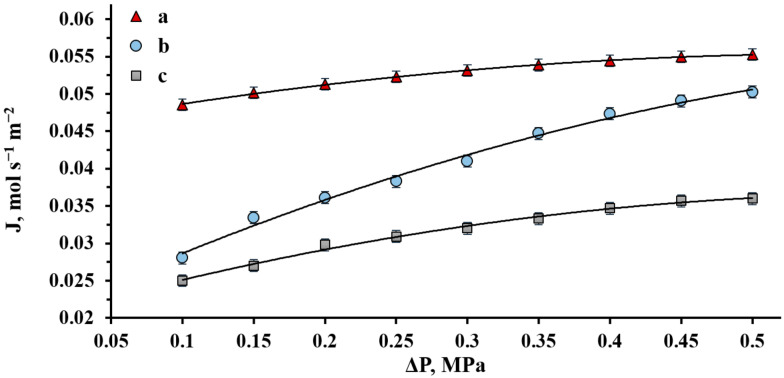
Gauge pressure dependence of the hydrogen flux at *t* = 300 °C on the inlet side of the PdCu–Nb–PdCu membrane modified with a nanostructured coating (a) and non-modified membrane (b) and a regular Pd membrane (c).

The data obtained during the first experiment step showed that the greatest increase in the hydrogen flux through the modified membrane was observed at a temperature of 300 °C. In this regard, the second experiment step was carried out at a selected constant temperature in the range of gauge pressures from 0.1 to 0.5 MPa (Figure 5). The hydrogen flux for the membrane modified with PdCu–Nb–PdCu at a gauge pressure of 0.5 MPa numerically reached values up to 0.055 mmol s^−1^ m^−2^. These data show that the hydrogen flux through a membrane with an applied functional layer can reach values 1.73 times higher than that through a membrane without such a layer at an optimal operating temperature of 300 °C.

As is known, the exponent *n* of the flux function is often used to indicate the stage responsible for controlling the hydrogen transport rate. Therefore, in the case of hydrogen transfer mechanism controlled by both surface processes and bulk diffusion, the value of the pressure index *n* changes from 0.5 (limiting by diffusion) to 1 (limiting by the surface) [31]. In the research, the exponent *n* for the membrane modified with PdCu–Nb–PdCu at 300 °C was 0.75. The obtained value indicates a significant effect of dissociative–associative processes on the hydrogen transport rate. Thus, it can be stated that permeability in operating conditions is limited not only by the transcrystalline transfer rate, but also by surface processes. This allows us to conclude that the application of a nanostructured modifying layer with such morphology accelerates dissociative–associative processes by an increase in the membrane surface’s development. Moreover, such membrane materials with an analogous protective modifying layer were investigated in methanol steam reforming and showed high kinetic characteristics of hydrogen transport as well as an absence of the negative influence from contact with a humid environment [32]. This confirms the possibility of using them in steam-reforming processes, which involve humid environment contact.

The selectivity of developed composite PdCu–Nb–PdCu membranes was investigated by the ratio of the hydrogen and nitrogen permeable fluxes at a gauge pressure in the retentate zone from 0.05 to 0.5 MPa and a constant temperature of 300 °C.

**Figure 6:**
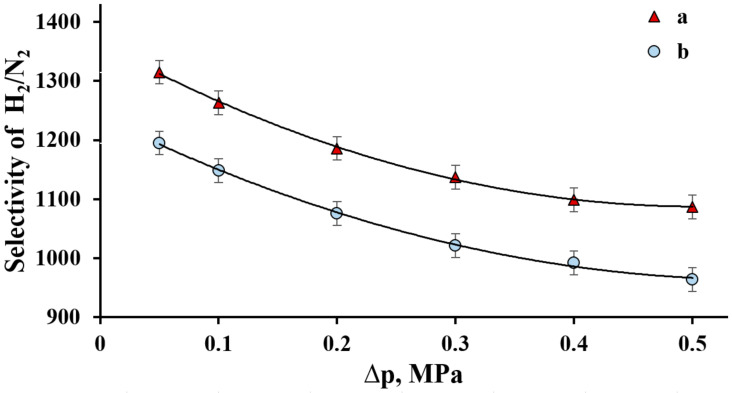
The gauge pressure dependence of the H_2_/N_2_ selectivity at 300 °C at the inlet side of PdCu–Nb–PdCu membrane modified with nanostructured coating (a) and non-modified membrane (b).

As shown in Figure 6, gauge pressure dependences of fluxes H_2_/N_2_ both modified and smooth composite PdCu–Nb–PdCu membranes demonstrated sufficiently similar selectivities at a pressure of 0.05 MPa. However, the greatest selectivity value was observed for modified membranes and reached values of up to 1323. According to the experiment results, the hysteresis dependence was not observed, which indicates the developed membranes’ ability to withstand pressure drops. The H_2_/N_2_ selectivity decrease to 1087 values was observed with a pressure increase on the membrane inlet side; nevertheless, the changes can be considered insignificant. This allows us to conclude that the Cu introduction into the protective layer did not affect the selective properties, and to judge the defect’s absence and the investigated membranes’ stability.

## 3. Materials and Methods

Pd-40%Cu alloy was deposited on both sides of a 50 μm niobium membrane by magnetron sputtering to create a thin protective layer. This reduced the cost of the obtained membranes and increased their durability, since hydrogen embrittlement was partially eliminated. The membrane surface was modified with a nanostructured catalytically active palladium layer by electrochemical deposition on a P-40X potentiostat-galvanostat (Ellins, Moscow, Russia). Composite PdCu–Nb–PdCu foil was preliminarily cleaned by washing in 96% ethanol and degreasing. Then, the prepared metal film was fixed on an inert holder made of silver with a purity of 99.99% and transferred to an electrolytic cell for modification. The holder was used as a current supply for the cathode. The PdCu–Nb–PdCu film was then transferred to a working cell, where it was first anodically polarized at a current density of 10–20 mA cm^−2^ in 0.1 M HCl and then cathodically polarized in 0.05 M H_2_SO_4_. Then, after washing with bidistillate, the electrochemical cell was filled with a growth solution of H_2_PdCl_4_ (2%) containing a surfactant tetrabutylammonium bromide (0.01 M). The nanostructured coating was deposited on both sides of the film at a current density of 4–5 mA cm^−2^. Finally, the modified foil was washed with bidistillate.

**Figure 7:**
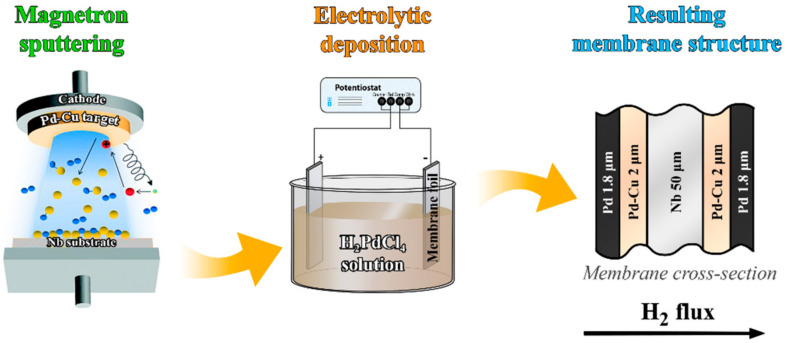
Stages of PdCu–Nb–PdCu membrane synthesis.

Stages of PdCu-Nb-PdCu membrane synthesis are shown in Figure 7.

Electron microscopy of the modified PdCu–Nb–PdCu films was carried out in the secondary electron (SE) mode on scanning electron microscope JEOL JSM-7500F.

Investigations on the kinetic characteristics of hydrogen transport of the obtained samples of modified composite PdCu–Nb–PdCu membranes were carried out on a hydrogen permeability unit, according to the method described in [33].

## 4. Conclusions

In this article, the modifying nanocrystalline palladium coating effect on the acceleration of the hydrogen transport through a composite PdCu–Nb–PdCu membrane was investigated. The influence of surface dissociative–associative processes on the rate of hydrogen transport through PdCu–Nb–PdCu membranes under operating conditions has been experimentally confirmed. A new approach to solving the problem of accelerating the surface stages of hydrogen transport has been reported, which consists of modifying the niobium membrane surface with a coating based on palladium nanocrystallites. The experiment’s results may indicate that the surface processes’ acceleration by increasing the membrane active surface area makes it possible to achieve an increase in the significant hydrogen flux by up to 1.73 times at a temperature of 300 °C. In addition, the modified PdCu–Nb–PdCu membrane is up to 2.5 times more permeable than a pure Pd membrane. Moreover, the developed composite membranes demonstrated high selectivity for H_2_/N_2_ values up to 1323. Thus, the modifying nanocrystalline palladium layer deposition, which significantly accelerates the surface stages of hydrogen transport through niobium membranes, makes it possible to create defect-free PdCu–Nb–PdCu membranes with high permeability at moderately low temperatures.

## Figures and Tables

**Table 1:** Statistical data of the surface morphology parameters of the PdCu–Nb–PdCu film modified with a nanostructured coating and non-modified film.

Film Type	Modified	Non-Modified
RMS roughness, nm	225.27	6.98
Mean roughness, nm	182.11	5.42
Roughness coefficient	16.18	1.54
Projected surface area, µm^2^	12	12
Actual surface area, µm^2^	194.12	18.48
